# Temporal trend in non-melanoma skin cancer mortality in China, 1992–2021: an analysis for the global burden of disease study 2021

**DOI:** 10.3389/fmed.2025.1495454

**Published:** 2025-05-14

**Authors:** Deng Li, Ge Yan, Shasha Song, Siqi Fan, Haochen Zhao, Gang Hu, Xuewen Xu, Qingfeng Li

**Affiliations:** ^1^Department of Plastic and Burns Surgery, West China Hospital, Sichuan University, Chengdu, China; ^2^School of Population Medicine and Public Health, Chinese Academy of Medical Sciences/Peking Union Medical College, Beijing, China; ^3^Department of Pathology, Yantai Fushan People’s Hospital, Yantai, China; ^4^Department of Urology, West China Hospital, Sichuan University, Chengdu, China; ^5^Health Manage Center, School of Health Management, Xinjiang Medical University, Urumqi, China; ^6^Department of Plastic and Reconstructive Surgery, Shanghai Ninth People’s Hospital Affiliated to Shanghai Jiao Tong University School of Medicine, Shanghai, China

**Keywords:** non-melanoma skin cancer, age-period-cohort, mortality, joinpoint regression analysis, Bayesian age-period-cohort, trends

## Abstract

**Introduction:**

Non - melanoma skin cancer (NMSC) is a widespread malignant neoplasm affecting the skin globally. In China, over the past 30 years, the prevalence and incidence of NMSC have changed significantly, yet mortality rate (MR) data is scarce. The aim is to assess the MR data of NMSC patients worldwide from 1992 to 2021, analyze its temporal trends, and provide valuable epidemiological information for future prevention and management strategies of NMSC.

**Methods:**

Using data from the Global Burden of Disease Study 2021 (GBD 2021), we analyzed crude mortality rate (CMR), age-standardized mortality rate (ASMR), and sex- and age-specific mortality trends, with temporal patterns assessed through longitudinal comparisons.

**Results:**

The MR for NMSC has shown an upward trend globally. From 1992 to 2021, both the CMR and ASMR for NMSC have increased substantially. The global ASMR has risen by approximately 30% during this period. Males have a higher ASMR compared to females, and the elderly population exhibits an accelerated and elevated ASMR trend for NMSC. In China, the mortality of NMSC is on the rise, with the current male MR exceeding that of females. Although the ASMR is projected to decline by 2030, the number of mortality cases is expected to increase, especially among males. The MR for NMSC shows a significant bias towards the elderly demographic.

**Discussion:**

The increasing mortality of NMSC, both globally and in China, highlights the importance of effective prevention and management strategies. In addition to implementing prevention and intervention measures in susceptible populations, it is crucial to establish a screening framework for NMSC to detect minor symptoms in a timely manner. This will help in early diagnosis and potentially reduce the mortality rate associated with NMSC. Thank you for your editorial support.

## Introduction

Non-melanoma skin cancer (NMSC) is a prevalent category of metastatic skin cancer, with basal cell carcinoma (BCC) and squamous cell carcinoma (SCC) comprising the most predominant types (1). BCC generally grows at a slow pace and presents a low risk of fatality and metastasis, although it has the potential to harm surrounding tissues (2). Clinically, BCC is identifiable by a painless, raised skin feature with a smooth texture, distinct minuscule blood vessels, or an ulcer at the elevated site (3). Contrarily, SCC possesses a greater tendency for metastasis and manifests as a hard nodule with a scaly exterior, potentially also ulcerating (4). NMSC ensues due to the cancerous transformation of irregular skin cells, leading to potential invasion and dissemination to various body parts (5). Given that NMSCs commonly originate in the skin’s epidermal layer, the tumors are often distinctly visible, facilitating the early detection of most NMSCs (6).

The incidence of NMSC has shown an escalating trend over recent years. This has resulted in significant economic burden for both patients and healthcare systems due to its high prevalence and increasing treatment costs (7). Improved understanding of risk factors might contribute to more effective prevention and early detection strategies, which could, in turn, help to mitigate this impact. While NMSC is not as lethal as melanoma, there are still a staggering 2–3 million new cases reported annually, indicating a high incidence rate (8). In 2020 alone, NMSC constituted 78% of all skin cancer cases, leading to an excess of 63,700 fatalities (9). Comparatively, in the same period, melanoma accounted for around 57,000 deaths. As of 2020, nearly 1.2 million NMSC cases were recorded globally (10).

The escalating incidence of NMSC represents a substantial public health burden in the 21st century, especially for low-to-middle income countries. The issue underlines the global severity of NMSC, underscoring the need for effective strategies to alleviate its impact and enhance patient quality of life. It is projected that the prevalence of NMSC in such countries will surge by 67% from 2010 to 2030 (11). Despite global health advancements resulting in increased life expectancy, NMSC remains a considerable threat to longevity (12). Additionally, NMSC prevalence is rising among younger age groups (13). China, as the largest developing nation, accommodates a substantial number of NMSC patients. Current statistics indicate that China has a population segment of 355 million aged 65 and older, projected to inflate to 543 million by 2030. The impending rise among China’s elderly population will inevitably intensify the burden of NMSC, presenting significant predicaments for public health and medical resources (14).

The principal objective of our research is to examine the temporal changes in mortality rates (MRs) of NMSC by applying a joinpoint regression model to analyze data from China spanning the years 1992–2021 (15). This model effectively deals with chronic disease data across different trend segments and exhibits efficient fitting results. The disparities in the MRs of NMSC between various genders and age groups were also analyzed, a factor crucial for the future prevention and management of the disease. Such comparisons aid in the identification of high-risk demographics and the creation of targeted intervention strategies.

## Materials and methods

### Data sources

The sample sources and technical data utilized were obtained from the Data Resources of the Global Burden of Disease Study 2021 (GBD 2021) (16). The estimation process for GBD identifies multiple data sources for each disease or injury. These sources extend from population censuses and vital statistics to disease registries and records of health service utilization, etc. (17). Identification of these data types is achieved via systematic reviews of published research, examination of governmental and published reports, primary data sources such as population health surveys, and datasets contributed by GBD collaborators (18).

A unique identifier is designated to each newly discovered and obtained data source, and it is subsequently included in the Global Health Data Exchange (GHDx) (19, 20). The GHDx permits public access to the metadata for each source in the GBD study, as well as the data itself, when permissible. Using the GHDx source tool, readers have the capability to discern the data sources used to produce estimations of disease or injury outcomes for any specific location. This study extracted age-specific mortality for NMSC spanning 1992–2021. GBD data were grouped into 5-year age intervals. Age-standardized mortality rates (ASMRs) were calculated using the World Standard Population as the reference. Log-transformation was applied to ASMRs in Joinpoint analysis. For BAPC models, raw death counts and population denominators were used directly. Data completeness precluded the need for imputation or additional smoothing (17).

### Joinpoint regression model

We analyzed temporal mortality trends in NMSC using joinpoint regression (Joinpoint Regression Program 4.9.10; National Cancer Institute) (21, 22). The model identifies segmented log-linear patterns in ASMRs, assuming homoscedastic residuals and temporal independence (23, 24). ASMRs (1992–2021), calculated via WHO standard population weights, were modeled as:


l⁢n⁢(γ)=α+β⁢xi+ϵ

where γ denotes mortality rate, x calendar year, and β_*i*_ the slope of each linear segment. Annual percentage change (APC) was derived as (e^β^*^i^*−1) × 100 (15). Model fitting employed weighted least squares regression on log-transformed ASMRs, with Monte Carlo permutation testing (10,000 iterations) determining optimal joinpoint configurations (maximum 5 joinpoints) and Bonferroni correction for multiple comparisons (25).

### Age-period-cohort model

The age-period-cohort framework disentangled temporal drivers of NMSC mortality through concurrent modeling of three dimensions: biological aging (age effects), population-wide healthcare shifts (period effects), and generational risk accumulation (cohort effects). Mortality rates were modeled as:


l⁢o⁢g⁢(M)=μ+α⁢i+β⁢j+γ⁢k+ϵ

where M represents mortality rates, μ denotes the intercept, and αi, βj, and γk parameterize age-specific susceptibility, period-derived healthcare advancements, and cohort-specific lifetime risk accumulation, respectively (26).

To resolve the inherent identifiability constraint, we applied the intrinsic estimator—a regularization approach that minimizes collinearity bias compared to conventional maximum likelihood estimation (27). This decomposition revealed inflection points where mortality trends deviated from historical trajectories, providing etiologically grounded insights for prevention strategies. The analysis was conducted using a web-based tool developed by the National Cancer Institute, USA.

### Bayesian age-period-cohort prediction model

We implemented Bayesian age-period-cohort (BAPC) modeling using the BAPC and INLA packages in R to project sex-specific NMSC mortality from 2020 to 2030. The model utilized Poisson-distributed death counts and population denominators stratified by 5-year age groups (0–4, …, 85+) and calendar years (1992–2021), with separate analyses for males and females (28). We assigned second-order random walk (RW2) priors to period and cohort effects and used weakly informative priors for their variances (29, 30). The model specification excluded external covariates to isolate age, period, and cohort dimensions:


$$log\left(E\left[D_{ijk}\right]\right)\;=\;log\left(P_{ijk}\right)\;+{\text{ α}}_{i}{\text{ + β}}_{j}\text{ + }{\text{γ}}_{k}$$

where D_*ijk*_ and P_*ijk*_ denote deaths and population counts for age group i, period j, and birth cohort k. Integrated nested Laplace approximations provided computationally efficient posterior sampling (31).

## Results

### Trends of age-standardized mortality and number of cases for non-melanoma skin cancer in China and globally, 1992–2021

**FIGURE 1:**
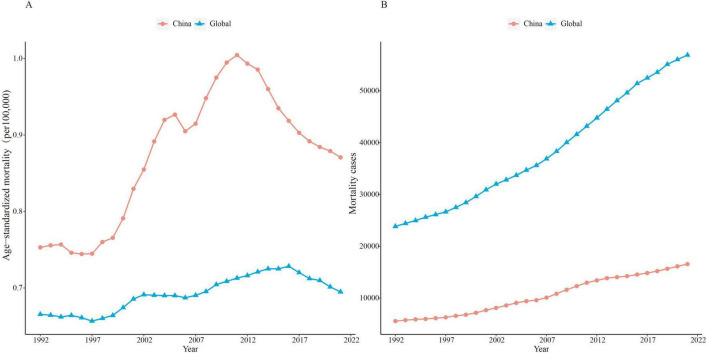
Trends in ASMR **(A)** and number of cases **(B)** of NMSC in China and the globe from 1992 to 2021.

For NMSC, from 1992 to 2021, China consistently reported higher ASMR than that in global. Specifically, the ASMR in China exhibited a distinct uptrend from approximately 0.7 per 100,000 per 100,000 in 1992 to over 1.0 per 100,000 at its peak in 2011 before experiencing a decline from 2012 to 2021. In contrast, the global ASMR has remained relatively stable over the same period (Figure 1). Regarding mortality cases, compared to the global trend, China’s mortality cases began at a much lower base of approximately 5,564 cases in 1992, showing a gradual increase, but the growth rate is relatively modest, reaching approximately 16,576 cases by 2021. The above analysis highlights a unique national trend that contrasts with the global trend and underscores the importance of understanding the temporal dynamics of NMSC mortality within the Chinese demographic.

### Trends in crude mortality and age-standardized mortality

Table 1 displays the CMRs and ASMR for NMSC at the outset (1992) and conclusion (2021) of the study period, along with the average annual percent changes (AAPCs) over 30 years and the annual percent changes (APCs) for each individual sub-period. A significant escalation was revealed through joinpoint analysis in the CMR of NMSC in China from 1992 to 2021, demonstrating an average annual expansion rate of 3.25% [95% confidence interval (CI) 3.06–3.44]. Since 1992, the CMR of NMSC for males heightened by +3.01% (95% CI 2.51–3.51) and for females by +3.43% (95% CI 3.23–3.62). Simultaneously, the AAPCs of NMSC MRs for male and female Chinese subjects approximated 3.25% (95% CI 3.06–3.44). Though a relatively stagnant upward pattern was observed between 1992 and 1998, with an APC of 1.93% yearly, a pronounced increase was noted between 1998 and 2004, with an APC of 5.33% yearly. However, the trend tapered off between 2004 and 2007, with an APC of 1.51% annually. Then a rising trend was evident between 2007 and 2011, with an APC of 6.22% yearly. In male subjects, despite the rising CMR of NMSC at an AAPC of 3.01% annually, a non-significant uptrend was detected between 1993 and 1996, with an APC of 0.3% annually. Nevertheless, a significant surge was observed from 1999 to 2003, with an APC of 6.32% annually. Female subjects experienced an upward growth in CMR of NMSC at an AAPC of 3.43% annually. This trend was particularly significant between 1998 and 2004 (APC: 5.08% annually), and between 2007 and 2011 (APC: 6.33% annually). The CMR for the overall population displayed an upward trend throughout the entire study duration, spanning from 1992 to 2021.

**TABLE 1:** Joinpoint analysis of crude mortality and age-standardized mortality in the Chinese population due to NMSC.

	MR (per 100,000)		Total study Period		Period 1		Period 2		Period 3		Period 4		Period 5		Period 6	
	**1992**	**2021**	**AAPC (%)**	**95% CI**	**Years**	**APC (%)**	**Years**	**APC (%)**	**Years**	**APC (%)**	**Years**	**APC (%)**	**Years**	**APC (%)**	**Years**	**APC (%)**
**Crude mortality**																
Both	0.46	1.16	3.25	(3.06, 3.44)	1992–1998	1.93*	1999–2004	5.33*	2005–2007	1.51*	2008–2011	6.22*	2012–2018	1.42*	2019–2021	2.54*
Female	0.44	1.60	3.43	(3.23, 3.62)	1992–1999	3.12*	2000–2004	5.08*	2005–2007	3.19*	2008–2011	6.33*	2012–2017	1.34*	2018–2021	2.40*
Male	0.48	1.17	3.01	(2.51, 3.51)	1992–1996	0.3	1997–1999	2.97	2000–2003	6.32*	2004–2007	3.08*	2008–2011	5.91*	2012–2021	1.64*
**Age-standardized mortality**																
Both	0.75	0.87	0.62	(0.42, 0.82)	1992–1999	1.00*	2000–2004	3.31*	2005–2007	0.16	2008–2011	2.77*	2012–2016	−2.20*	2017–2021	−1.11*
Female	0.64	0.77	0.62	(0.42, 0.82)	1992–1999	1.00*	2000–2004	3.31*	2005–2007	0.16	2008–2011	2.77*	2012–2016	−2.20*	2017–2021	−1.11*
Male	0.93	1.03	0.32	(−0.23, 0.88)	1992–1996	−1.78*	1997–1999	0.64	2000–2003	4.50*	2004–2012	1.22*	2013–2018	2.11*	2019–2021	−0.36

APC values marked with an asterisk (*) indicate statistically significant trends at *p* < 0.05.

Since 1992, the ASMR for NMSC in China has shown an annual increase of 0.62%, with the rate adjusted for the population’s age distribution (95% CI 0.42–0.82). This trend encompasses three distinct periods of change—an initially modest increase of 1.00% between 1992 and 1999, a substantial increase of 3.31% from 1999 to 2004, a slight growth of 0.16% from 2004 to 2007, and two significant increments of 2.77% and 2.20% from 2007 to 2011 and 2011–2016, respectively (each *p* < 0.05), followed by a moderate rise post-2016. The trend for women is the same as for the general population. Inspection of male-specific mortality trends uncovers four stages in NMSC mortality. There was a flat growth of 1.78% and 0.64% from 1992 to 1996 and 1996–1999 respectively, a significant increase of 4.50% from 1998 to 2003, a modest increase of 1.22% and 2.11% from 2003 to 2012 and 2012–2018, and a further slight increase of 0.36% post-2018 (each *p* < 0.05).

**FIGURE 2:**
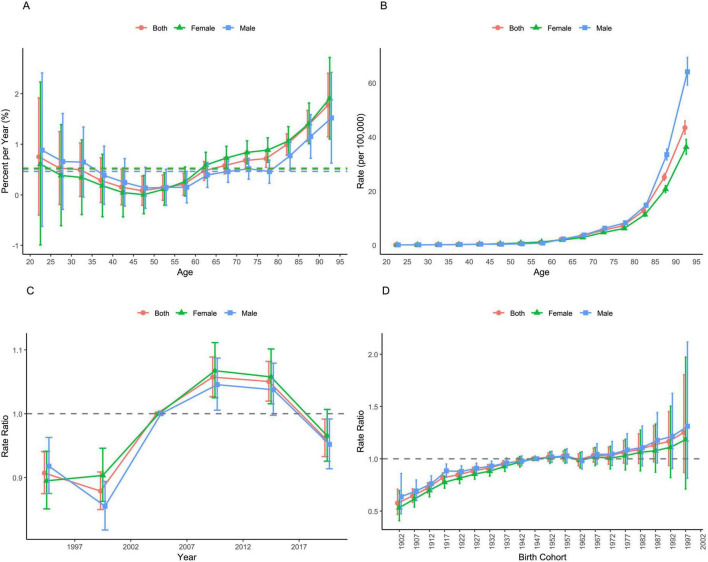
Age-period-cohort effects on MRs of NMSC in China from 1992 to 2021. **(A)** Local and net drift in MRs of NMSC; **(B)** age effect on MRs of NMSC; **(C)** period effect on MRs of NMSC; **(D)** cohort effect on MRs of NMSC.

Figure 2A shows the local and net drift in MRs of NMSC. MRs decrease in the age groups up to 45–50 years, then rise thereafter, with a marked acceleration after the 80–85 age group. Males have higher MRs than females, but starting from the 50–55 age group, male MRs fall below female MRs. The sharp increase in MRs after 80–85 years highlights the heightened risk in older age groups.

Figures 2B–D illustrates the age, period, and cohort effects on the MR of NMSC. Post period effect adjustments, the NMSC MR exhibits an upward trend with age, significantly escalating in older age brackets. Moreover, males consistently display a higher MR. Period effects reveal an upward trend in NMSC MR for female, and a downward trend for male from 1992 to 1999, followed by an increasing trend for both genders from 1999 to 2009, and subsequently a declining trend from 2009 to 2019, especially, females demonstrate a higher MR from 1999 to 2019. Cohort effects suggest an increasing risk in most subgroups for cohorts born between 1902 and 1959. Conversely, cohorts born between 1959 and 1975 exhibit a decreasing risk in most subgroups. For the cohorts born from 1975 to 1999, the risk rises consistently in most subgroups.

### Trends in age-specific mortality rate

Over the course of the study, NMSC’s ASMR failed to exhibit significant alterations within the 30–44 age bracket in Chinese adults. However, variations were noted in other age groups, notably an appreciable increase in ASMR among those aged 55 and above. It’s significantly augmented in the 20–24, 24–29, 30–34, 35–39, and 55 and older demographics during the study. Notably, among females, the ratios within the 20–24, 25–29, 30–34, 35–39, and 40–44 age brackets demonstrated a significant decline, with corresponding APC levels of 0.6% (95% CI −1.0, 2.2), 0.4% (95% CI −0.6, 1.4), 0.3% (95% CI −0.4, 1.1), 0.2% (95% CI −0.4, 0.8), and 0.0465% (95% CI −0.4, 0.5), respectively. Conversely, between the 45 and 50 and 85+ age groups, substantial increases were noted with APC values standing at 0.0061% (95% CI −0.4, 0.4), 0.1% (95% CI −0.2, 0.4), 0.3% (95% CI −0.0199, 0.6), 0.6% (95% CI 0.3, 0.8), 0.7% (95% CI 0.5, 1.0), 0.8% (95% CI 0.6, 1.1), 0.9% (95% CI 0.6, 1.1), 1.1% (95% CI 0.8, 1.3), 1.4% (95% CI 1.0, 1.8), and 1.9% (95% CI 1.1, 2.7) respectively. For males, noticeable increases were noted in the 75–79, 80–84 and 85+ age ranges, with APC levels of 0.5% (95% CI 0.2, 0.7), 0.8% (95% CI 0.5, 1.0), 1.2% (95% CI 0.7, 1.6), and 1.5% (95% CI 0.6, 2.4), respectively (Table 2). This data suggests a rising NMSC MR among Chinese seniors in recent decades.

**TABLE 2:** APCs in age-specific MRs of NMSC.

Group	Age	Percent per year	CI lower	CI upper	Low	High
Both	20–24	0.7527	−0.3968	1.9155	1.1495	1.1628
Both	25–29	0.5278	−0.1887	1.2495	0.7165	0.7217
Both	30–34	0.4971	−0.0287	1.0257	0.5258	0.5286
Both	35–39	0.2889	−0.1496	0.7294	0.4385	0.4405
Both	40–44	0.1552	−0.1941	0.5057	0.3493	0.3505
Both	45–49	0.0842	−0.2047	0.3739	0.2889	0.2897
Both	50–54	0.1444	−0.1038	0.3931	0.2482	0.2487
Both	55–59	0.2098	−0.0086	0.4287	0.2184	0.2189
Both	60–64	0.4735	0.2898	0.6576	0.7633	0.1841
Both	65–69	0.5774	0.415	0.7401	0.9924	0.1627
Both	70–74	0.6787	0.5189	0.8387	1.1976	0.16
Both	75–79	0.7159	0.5428	0.8894	1.2587	0.1735
Both	80–84	0.9939	0.7833	1.2049	1.7772	0.211
Both	85–89	1.3605	1.0526	1.6693	2.4131	0.3088
Both	90–94	1.7736	1.1465	2.4046	2.9201	0.631
Female	20–24	0.6059	−0.9917	2.2292	1.5976	1.6233
Female	25–29	0.3853	−0.6069	1.3874	0.9922	1.0021
Female	30–34	0.3462	−0.3875	1.0852	0.7337	0.739
Female	35–39	0.1835	−0.4314	0.8021	0.6149	0.6186
Female	40–44	0.0465	−0.4339	0.5293	0.4804	0.4828
Female	45–49	0.0061	−0.3723	0.3858	0.3784	0.3797
Female	50–54	0.1162	−0.2028	0.4362	0.319	0.32
Female	55–59	0.2651	−0.0199	0.5508	0.285	0.2857
Female	60–64	0.5878	0.3355	0.8408	0.9233	0.253
Female	65–69	0.7278	0.4972	0.959	1.225	0.2312
Female	70–74	0.8383	0.6102	1.067	1.4485	0.2287
Female	75–79	0.8841	0.6421	1.1266	1.5265	0.2425
Female	80–84	1.0643	0.7823	1.3471	1.8466	0.2828
Female	85–89	1.4117	1.0103	1.8147	2.422	0.403
Female	90–94	1.906	1.1024	2.7161	3.0084	0.8101
Male	20–24	0.8818	−0.6229	2.4092	1.5047	1.5274
Male	25–29	0.6567	−0.2862	1.6086	0.9429	0.9519
Male	30–34	0.6464	−0.0422	1.3397	0.6886	0.6933
Male	35–39	0.3873	−0.185	0.9628	0.5723	0.5755
Male	40–44	0.2461	−0.2201	0.7145	0.4662	0.4684
Male	45–49	0.1387	−0.2662	0.5451	0.4049	0.4064
Male	50–54	0.1491	−0.206	0.5055	0.3551	0.3564
Male	55–59	0.1533	−0.1553	0.4629	0.3086	0.3096
Male	60–64	0.3952	0.1496	0.6413	0.5448	0.2461
Male	65–69	0.4595	0.2496	0.6698	0.7091	0.2103
Male	70–74	0.5158	0.3105	0.7216	0.8263	0.2058
Male	75–79	0.459	0.232	0.6866	0.691	0.2276
Male	80–84	0.7726	0.4849	1.0611	1.2575	0.2885
Male	85–89	1.1529	0.722	1.5856	1.8749	0.4327
Male	90–94	1.5194	0.6246	2.4222	2.144	0.9028

“CI lower” and “CI upper” represent the 95% confidence interval bounds for the APC. “Low” and “high” denote the minimum and maximum values within the observed range.

### Predictions of trends in age-standardized mortality and mortality cases from 2022 to 2030

**FIGURE 3:**
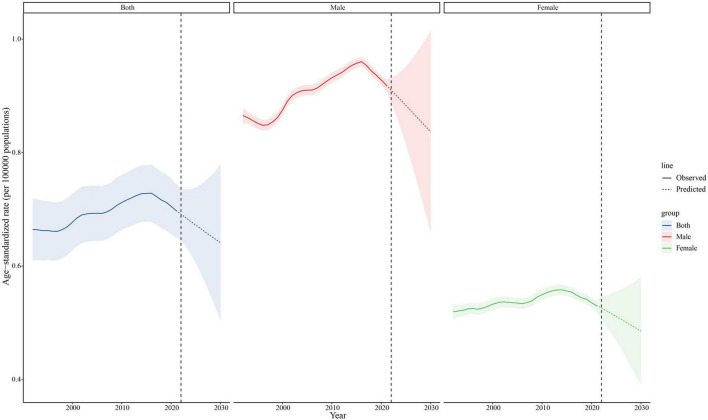
Trends in ASMR by both genders, males and females predicted by the BAPC models, 1992–2030.

**FIGURE 4:**
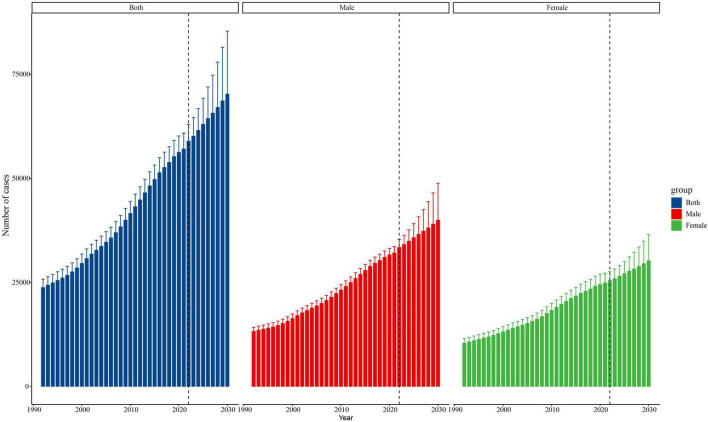
Trends in the number of mortality cases by both sexes, males and females predicted by the BAPC models, 1992–2030.

According to the BAPC model, the ASMR for the total population is anticipated to experience a gradual decline from 2022 to 2030 (Figure 3). Specifically, females are projected to see a substantial and steep decrease in their ASMR by approximately 9.44%, reducing from 5,332 in 2022 to 4,829 by 2030. In contrast, the model forecasts a more gradual decline for males, with their ASMR decreasing by 5.45%, dropping from 7,538 in 2022 to 7,128 by 2030. Despite these promising trends in ASMR for both genders, the total number of mortality cases is expected to continue rising. As depicted in Figure 4, the number of mortality cases is projected to increase by 24.90%, demonstrating an accelerating trend from 17,414 cases in 2022 to an estimated 21,750 cases by 2030. This uptrend is particularly pronounced in males, with a projected increase of 28.13%, reaching 11,848 cases by 2030. Additionally, females also exhibit a continuous and stable increase amounting to 21.25% in the number of cases within this timeframe, which is expected to reach 9,901 by 2030, albeit at a lower rate than males.

## Discussion

To our knowledge, this represents the inaugural nationwide analysis of NMSC burden in China spanning the years 1992–2021. Utilizing mortality statistics from the GBD study, we examined the NMSC mortality patterns across various age and gender demographics over 3 decades. Our findings indicate a consistent increase in the MR of NMSC in the older population, irrespective of gender. Further, we found that even though the ASMR was consistently lower in females compared to males throughout the study duration, the rate of increase among females was larger, resulting in a narrowing disparity between the two groups.

In the GBD study, the MR of NMSC shows significant variation worldwide. Although data on skin cancer trends in Asian countries is somewhat sparse, reports detailing alteration in melanoma and non-melanoma incidence rates in Singapore (32, 33), Hong Kong, China (34), and Japan (35) are available. It’s noteworthy that these rates exhibit different tendencies based on geographical location and race, even within the Asian demographic (36–38). For instance, in Spain, the MR associated with NMSC dropped by 48.9%, from 2.32 per 100,000 residents (95% CI: 2.24–2.77) in 1979 to 1.13 per 100,000 residents (95% CI: 1.16–1.39) in 2019 (39). Meanwhile, in the North Rhine-Westphalia region of Germany, the estimated NMSC MR per 100,000 individuals rose from 0.17 in 2007 to 0.9 in 2016. In contrast, Australia’s estimated NMSC MR remained fairly consistent at around 2.0–2.1 from 2007 to 2016 (40). Like the U.S., North Rhine-Westphalia and Australia’s populations are predominantly Caucasian. GBD data reveal that over the past 3 decades, the U.S.’s NMSC MR has remained rather steady at 0.74 in 1990 and 0.76 in 2019 (41). One study predicted that the total number of NMSC related deaths in the U.S. ranged from 3,900 to 8,800 in 2012 (42), in alignment with the respective GBD figures of 3,728 in 2012 and 4,472 in 2019.

Our investigation revealed that the basic MR for NMSC in China experienced an increase from 0.46 per 100,000 inhabitants in 1992 to 1.16 per 100,000 inhabitants in 2021. Consequentially, there has been a 152.2% increase in the CMR of NMSC in China over the past 3 decades. Regarding the ASMR of NMSC, the global ASMR (per 100,000 population) for NMSC ranged from 0.5 to 0.5 between the years 1990 and 2010 (43). On the other hand, the ASMR for NMSC in China (per 100,000 population) ranged from 0.75 to 0.87 between 1992 and 2021, surpassing the global average by 50–74%. This highlights the urgent need for addressing the issue of NMSC in China.

NMSCs often possess ultraviolet (UV) signature mutations, which suggests that UV radiation, through direct DNA damage, is a causal factor for these cancers (44). The MR of NMSC in China is experiencing a rapid increase, whereas the MR of NMSC in the United States has remained relatively stable. This could potentially be due to advanced medical facilities in the United States or greater emphasis on prevention and treatment. Germany, located in a region with higher average latitude and thereby lower average UV intensity, has a lower MR of NMSC as compared to China. However, the MR of NMSC in China remains less than that in Spain and Australia.

Chronic exposure to UV radiation negatively impacts the functionality and survival of various cell types in human skin and is widely recognized as the primary causative agent of skin cancer (45, 46). Elevated MRs from NMSC seen in countries like Australia and Spain could be driven by external variables such as altitude and light intensity. The accelerated surge in NMSC’s MR in China is potentially the result of factors like ionizing radiation, pollutants, chemicals, and occupational exposure. Data from the Surveillance, Epidemiology, and End Results program in the United States verify that NMSC incidence is rising irrespective of socio-economic status, possibly due to environmental exposures such as sunlight or tanning (41). However, contrary to Western countries, the Chinese population does not follow a trend of increased sun exposure. Thus, the surge in NMSC within China is likely attributable to heightened skin cancer awareness in the public sphere and the widespread use of punch biopsy tools in the country, even though the absolute increase in NMSC remains minimal. Evidence from a population-based study in Germany (the SCREEN project) suggests skin cancer screenings could heighten NMSC incidence (7). However, the observed decrease in NMSC’s MR in China could be attributed to enhancements in medical facilities and the widespread implementation of early cancer screening measures, eventually leading to a gradual reduction in NMSC’s MR.

Our study revealed that between 1992 and 2021, the MR of NMSC was markedly higher among Chinese men than among Chinese women. Specifically, the MR of NMSC in males escalated from 0.48 per 100,000 population in 1992 to 1.17 in 2021. Concurrently, data from the GBD database underscored an uptick in the ASMR of NMSC in China, rising from 0.75 per 100,000 population in 1992 to 0.87 in 2021. In women, the ASMR also experienced a surge, from 0.64 per 100,000 population in 1992 to 0.77 in 2021. Similarly, for men, the MR of NMSC witnessed an upward trend from 0.93 in 1992 to 1.03 in 2021. Notably, men had significantly elevated MR of NMSC compared to women between 1992 and 2021. Potential explanations for such differences may include variations in sex hormone genetics, disparities in adaptive and congenital immune responses, as well as variances in antioxidant stress resistance capabilities (47). These trends could also partially be attributed to sex-linked discrepancies in healthcare practices, such as men’s lower participation rates in cancer screening (48).

The evaluation of age-period-cohort effects revealed a reduced risk of mortality from NMSC amongst younger age groups and more recent birth cohorts. The increasing trend in ASMR for NMSC across all elderly population groups in China, alongside enhanced risks across varying time periods and birth cohorts, suggest marked potential for improvement in NMSC prevention and treatment efforts in China. This point is especially critical given the country’s rapidly aging population.

The declining trend in MRs of NMSC among Chinese men and women post-2011 may reflect enhancements in healthcare provision and disease prevention strategies within China. Environmental factors have also proven to exert influence on NMSC incidences (49, 50). To continue the downward trajectory of NMSC MRs and accomplish the objectives set forth in the “Healthy China 2030” blueprint, curbing environmental pollution is essential. The relatively consistent trend of ASMR of NMSC in urban China from 2012 to 2019 implies the interplay of multiple factors. The Environmental Protection Law of the People’s Republic of China, enacted from January 1, 2015, onward, likely played a role in reducing ASMR of NMSC after 2016.

The ASMR for NMSC within China’s elderly population exhibited a rising trend throughout the duration of our study. This outcome underscores the necessity for heightened focus on NMSC in older individuals.

The BAPC analysis indicated that the number of mortality cases is expected to continue rising, albeit a promising decline in ASMR for both genders has been observed post-2012. This trend aligns with global patterns where public health efforts have effectively reduced mortality rates. However, the continued increase in mortality cases underscores demographic shifts—mainly population growth and aging—as critical factors. This discrepancy highlights the unique demographic pressures China faces compared to other nations. For males, who exhibit a more pronounced rise in projected mortality cases, targeted interventions such as occupational health measures for high UV exposure areas are crucial. Gender-specific health campaigns and regular screenings are essential for early detection and effective management.

Generally, the variations in CMRs and ASMRs for skin cancer across different countries can be attributed to demographic factors such as age, gender, ethnicity, duration of outdoor activities, use of sunscreen, and health insurance coverage. Country-specific features, like UV index, climate, healthcare accessibility, the portion of the national budget devoted to medical services, and shifts in population composition, also play a significant role in explaining these disparities.

This study possesses several limitations. Initially, the data gathered from the GBD consists of estimations from multiple sources and mathematical models, meaning not all types of skin cancer may be accounted for. Also, potential information bias is an issue due to the relative inaccuracy of data available across various countries. Other limitations associated with the GBD database have been discussed elsewhere in the literature. In addition, the joinpoint regression model did not incorporate covariates or account for confounding factors affecting the outcome variable. Lastly, the absence of microdata on NMSC cases constrains the comprehensiveness of the analysis.

## Conclusion

The MR of NMSC in China is escalating. An accelerated increase in this MR has caused a contraction in the gender disparities, now with males disclosing higher MRs compared to females. A larger incidence of these MRs is observed within the elderly population. As China’s demographic continues to age, the impact of death and disability resulting from NMSC and its related complications will persistently grow. Therefore, developing preventive and intervention strategies is crucial. Such measures could include the establishment of surveillance frameworks for early NMSC detection, intensifying interventions and rehabilitation for NMSC patients, and promoting healthier lifestyles.

## Data Availability

The datasets presented in this study can be found in online repositories. The names of the repository/repositories and accession number(s) can be found at: https://ghdx.healthdata.org/gbd-2021.
